# Molecular hydrogen protects against ischemia-reperfusion injury in a mouse fatty liver model via regulating HO-1 and Sirt1 expression

**DOI:** 10.1038/s41598-018-32411-4

**Published:** 2018-09-19

**Authors:** Shaowei Li, Masayuki Fujino, Naotsugu Ichimaru, Ryosuke Kurokawa, Shinichi Hirano, Lisha Mou, Shiro Takahara, Terumi Takahara, Xiao-Kang Li

**Affiliations:** 1 Division of Transplantation Immunology, National Research Institute for Child Health and Development, Tokyo, Japan; 2 Third Department of Internal Medicine, University of Toyama, Toyama, Japan; 3 Department of Advanced Technology for Transplantation, Osaka University Graduate School of Medicine, Osaka, Japan; 4 AIDS Research Center, National Institute of Infectious Diseases, Tokyo, Japan; 5 MiZ Co., Ltd., Kanagawa, Japan; 6 Shenzhen Domesticated Organ Medical Engineering Research and Development Center, Shenzhen Second People’s Hospital, First Affiliated Hospital of Shenzhen University, Shenzhen, China

## Abstract

Fatty liver has lower tolerance against ischemia-reperfusion (I/R) injury in liver operations, including liver transplantation. Seeking to ameliorate liver injury following I/R in fatty liver, we examined the protective effect of hydrogen (H_2_) saline on I/R liver injury in a methionine and choline-deficient plus high fat (MCDHF) diet-induced fatty liver mouse model. Saline containing 7 ppm H_2_ was administrated during the process of I/R. Livers were obtained and analyzed. Primary hepatocytes and Kupffer cells (KCs) were obtained from fatty liver and subjected to hypoxia/reoxygenation. Apoptosis-related proteins and components of the signaling pathway were analyzed after treatment with hydrogen gas. The MCDHF I/R group showed higher levels of AST and ALT in serum, TUNEL-positive apoptotic cells, F4/80 immunopositive cells, mRNA levels of inflammatory cytokines, constituents of the signaling pathway, pro-apoptotic molecules in liver, and KCs and/or primary hepatocytes, compared to the control group. In contrast, H_2_ treatment significantly suppressed the signs of I/R injury in fatty liver. Moreover, the expression of Bcl-2, HO-1, and Sirt1 in liver, KCs, and hepatocytes by hydrogen gas were increased, whereas caspase activation, Bax, and acetylation of p53 were suppressed by hydrogen gas. These results demonstrated that H_2_ treatment ameliorated I/R liver injury in a fatty liver model by reducing hepatocyte apoptosis, inhibiting macrophage activation and inflammatory cytokines, and inducing HO-1 and Sirt1 expression. Taken togather, treatment with H_2_ saline may have a protective effect and safe therapeutic activity during I/R events, such as in liver transplantation with fatty liver.

## Introduction

The shortage of organs demands the use of “suboptimal grafts” and within the Eurotransplant community approximately 15,000 patients were listed for organ transplantation of which 1853 were awaiting orthotopic liver transplantation (OLT) (Eurotransplant Statistics, 2014). To overcome limitations in graft allocations, the extended criteria for donor (ECD) selection were developed by considering donor age, hypernatremia, prolonged cold ischemia time, and macrovesicular steatosis^1^. Macrosteatosis significantly impairs the tolerance for hepatic I/R injury^2^. Liver I/R activates leukocytes, endothelial cells, and Kupffer cells (KCs) and makes them interact, releasing tumor necrosis factor (TNF) that triggers the cascade of damage^3^. As key mediators of the immune response, KCs participate in hepatic I/R injury^4^.

Recently, some researchers have found that molecular hydrogen has potent pharmacological effects via reducing oxidative stress, inflammation, and apoptosis^5–7^. Molecular hydrogen has emerged in the form of hydrogen-rich water, or inhaled hydrogen gas, and is recommended as an effective treatment for cardiac arrest in clinical applications (HYBRID II TRILA; Ministry of Health, Labour and Welfare of Japan, No. 68; 2017). There are many way to administrate molecular hydrogen; among them oral treatment is the most convenient, but some hydrogen gas will escape into the stomach. Administration of hydrogen saline to target tissue allows the molecular hydrogen delivery to be more efficient^8^. The latest report derived from rat models has opened a novel, promising therapeutic application of image-guided hydrogen bubble delivery to prevent myocardial ischemia–reperfusion injury^9^.

Heme oxygenase-1 (HO-1), a rate-limiting enzyme catalyzing the conversion of heme into biliverdin, carbon monoxide, and iron, plays an important role in anti-oxidative and anti-inflammatory effects. Our preliminary experiment indicated the protective effects of HO-1 expression by 5-aminolevulinic acid combined with ferrous iron on lipopolysaccharide (LPS)-stimulated macrophages (unpublished data). Numerous studies have demonstrated that Sirtuin 1 (Sirt1), a member of the sirtuin family, is not only an NAD^+^ dependent type III histone and protein deacetylase, but also a kind of long-lived gene that inhibits apoptosis and improves cell survival in mammalian cells^10–12^. Sirt1 often acts as a transcriptional switch to participate in regulating many substrates in the pathogenesis of many diseases (similarly to p53, an enzyme involved in DNA repair)^13^. Currently, it is known that HO-1 has a cyto-protective effect through Sirt1 to form the functional module^14^. These helpful data validated the potential capacity of Sirt1 to be involved in the improvement of various diseases. Recently, it has been reported that hydrogen molecules could ameliorate hyperoxia-induced lung injury related to endoplasmic reticulum (ER) stress and that the mechanism of hydrogen’s effect may be associated with upregulation of Sirt1^15^.

The aim of the present study was to determine whether molecular hydrogen had a protective effect against I/R injury by enhanced HO-1 and Sirt1 expression and to explore the role of the HO-1/Sirt1 axis in the apoptosis of fatty primary hepatocytes after hypoxia/reoxygenation.

## Results

### Fatty livers subjected to I/R exhibited liver injury, macrophage infiltration, hepatocyte apoptosis, and oxidative stress, which were suppressed by hydrogen saline

**Figure 1:**
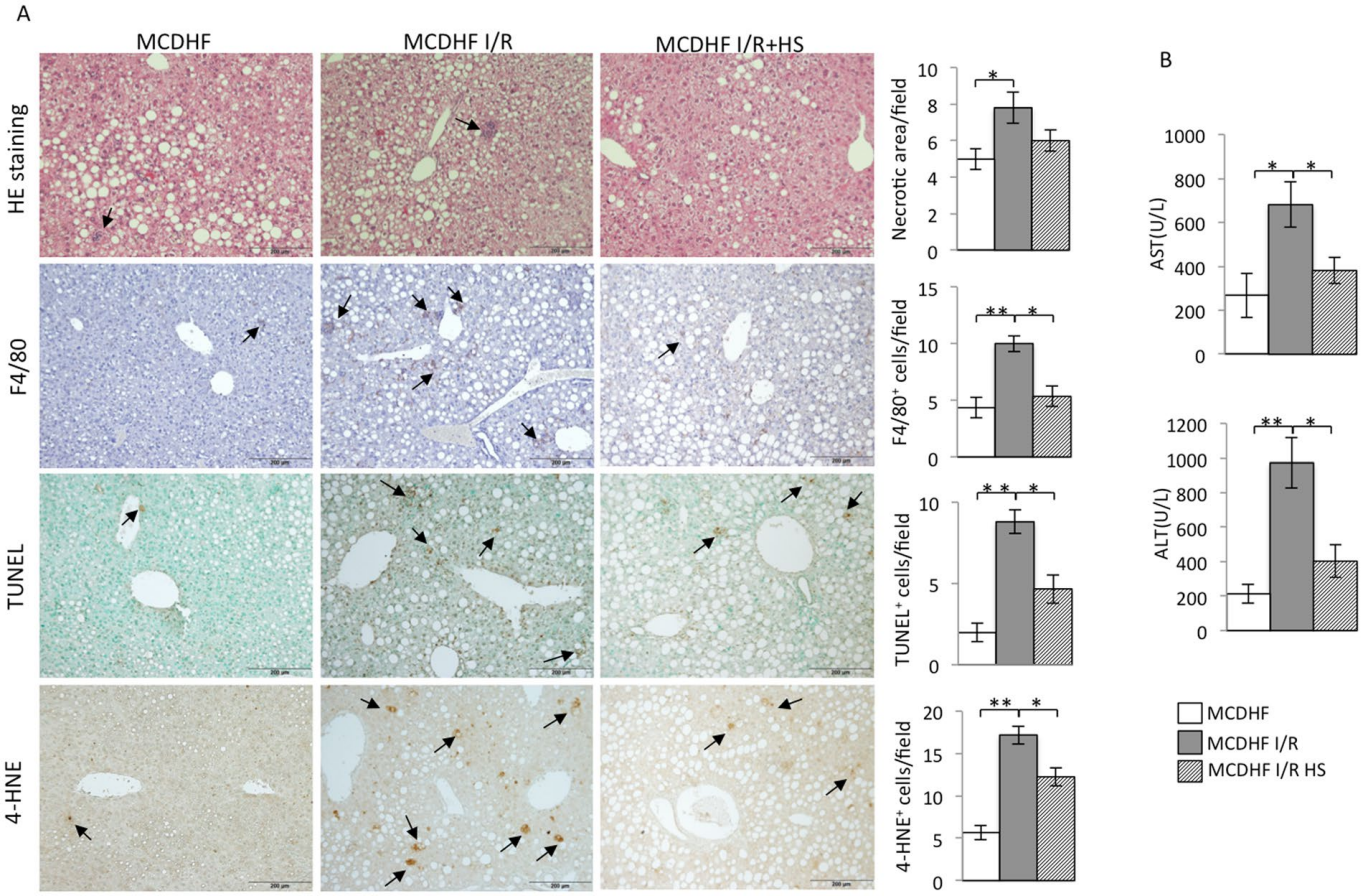
Hydrogen saline attenuated necrosis, infiltration of macrophages, oxidant stress, and apoptosis as well as the biochemical parameters related to I/R injury in the steatotic liver. (**A**) The necrotic area (first line) of the MCDHF diet group (left panel), I/R group (middle panel), and hydrogen saline treatment group (right panel), TUNEL-positive cells (arrows, second line), F4/80-positive cells (arrows, third line), and 4-HNE-positive cells (arrows, fourth line). Scale bars represent 200 μm. (**B**) The number of necrotic areas, TUNEL-positive cells, F4/80-positive cells, and 4-HNE-positive cells and the serum ALT and AST levels after 15 minutes ischemia and 3 hours reperfusion. Data are expressed as the means ± SEM; *p < 0.05, **p < 0.01.

The serum levels of AST & ALT were elevated within 3 hr reperfusion following 15 min ischemia in steatotic liver, while the group with hydrogen saline administration effectively decreased the AST and ALT levels compared to the control group (Fig. 1B). Hydrogen saline could prevent histological alterations (necrosis). The histology of the steatotic livers showed that there were almost no inflammatory cells or necrotic areas in the MCDHF diet group, whereas inflammatory cell infiltration and necrotic areas were found after 3 hr reperfusion and 15 min ischemia (Fig. 1A). Necrotic areas of the steatotic liver were analyzed to determine the preventive effect of hydrogen saline treatment. Hydrogen saline pretreatment decreased the liver histological damage when subjected to I/R injury (Fig. 1A). The number of F4/80-positive cells, macrophages, was more widely distributed in the group with 3 hr reperfusion after 15 min ischemia than in control fatty liver. In contrast, pre-treatment with hydrogen saline significantly decreased the population of mouse EGF-like module-containing mucin-like hormone receptor-like 1 (EMR1 or F4/80) positive cells (Fig. 1B). After 3 hr reperfusion following 15 min ischemia, the amount of TUNEL-positive cells was significantly increased compared to fatty liver. Hydrogen saline administration dramatically suppressed the amount of TUNEL-positive cells (Fig. 1A). Moreover, the number of cells positive for 4-hydroxynonenal(4-HNE), product of lipid peroxide, was significantly decreased in the hydrogen saline treatment group, indicating that hydrogen saline protected hepatic parenchymal cells from I/R injury-induced peroxidation.

### Hydrogen saline pretreatment induced HO-1 & Sirt1 expression in steatotic livers subjected to I/R injury

**Figure 2:**
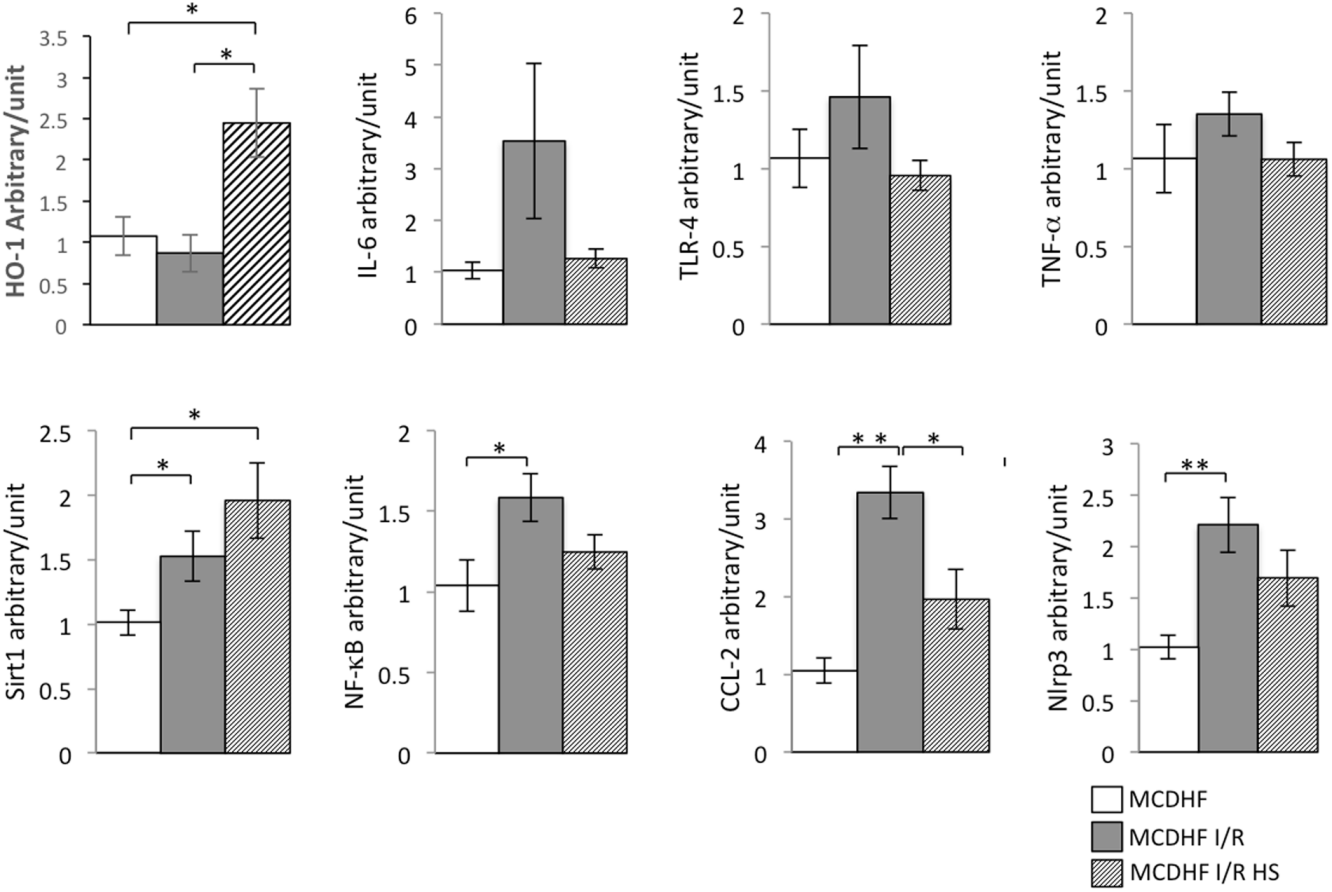
Hydrogen saline decreased the expression of inflammatory cytokines and the biochemical parameters, but increased the Sirt1 and HO-1 expression in the steatotic liver under I/R injury. The mRNA expression of TNF-α, IL-6, NF-κB, TLR-4, CCL-2, Nlrp3, Sirt1, and HO-1 in different groups. (Data are representative of 5 to 7 animals for each group). Data are expressed as the means ± SEM; *p < 0.05, **p < 0.01.

In numerous studies, the high expression levels of HO-1 & Sirt1 can alleviate the ischemic injury. Analogously, our results showed that mRNA levels of HO-1 was dramatically upregulated in steatotic livers pretreated hydrogen saline and subjected to 15 min ischemia and 3 hr reperfusion compared with steatotic livers and steatotic livers subjected to I/R injury at the same time, as shown in Fig. 2. The expression of Sirt1 was also significantly increased in steatotic livers pretreated hydrogen saline and subjected to 15 min ischemia and 3 hr reperfusion compared with steatotic livers subjected to I/R injury, moreover, compared with the steatotic livers treated with sham operation.

### mRNA expression of inflammatory cytokines reduced by hydrogen saline in steatotic livers subjected to I/R

We evaluated the expression of inflammatory cytokines by detecting the following indicators: tumor necrosis factor α (TNF-α), interleukin-6 (IL-6), nuclear factor kappa B (NF-κB), toll-like receptor 4 (TLR-4) and NOD-like receptor 3 (Nlrp3). In contrast to fatty liver sham operation group, there was a marginally (TNF-α and IL-6) or significantly (TLR-4 and Nlrp3) higher level of expression of all of the above factors by evaluating mRNA in the steatotic livers following 3 hr reperfusion after 15 min ischemia (Fig. 2). In the hydrogen saline-treatment group the expressions of the above factors were reduced, moreover, monocyte chemoattractant protein-1 (MCP-1/CCL2) was down-regulated significantly.

### Hydrogen saline regulated inflammatory cytokines, HO-1, Sirt1, TLRs, and nuclear translocation of NF-κB mRNA expression in KCs isolated from steatotic livers subjected to I/R injury

**Figure 3:**
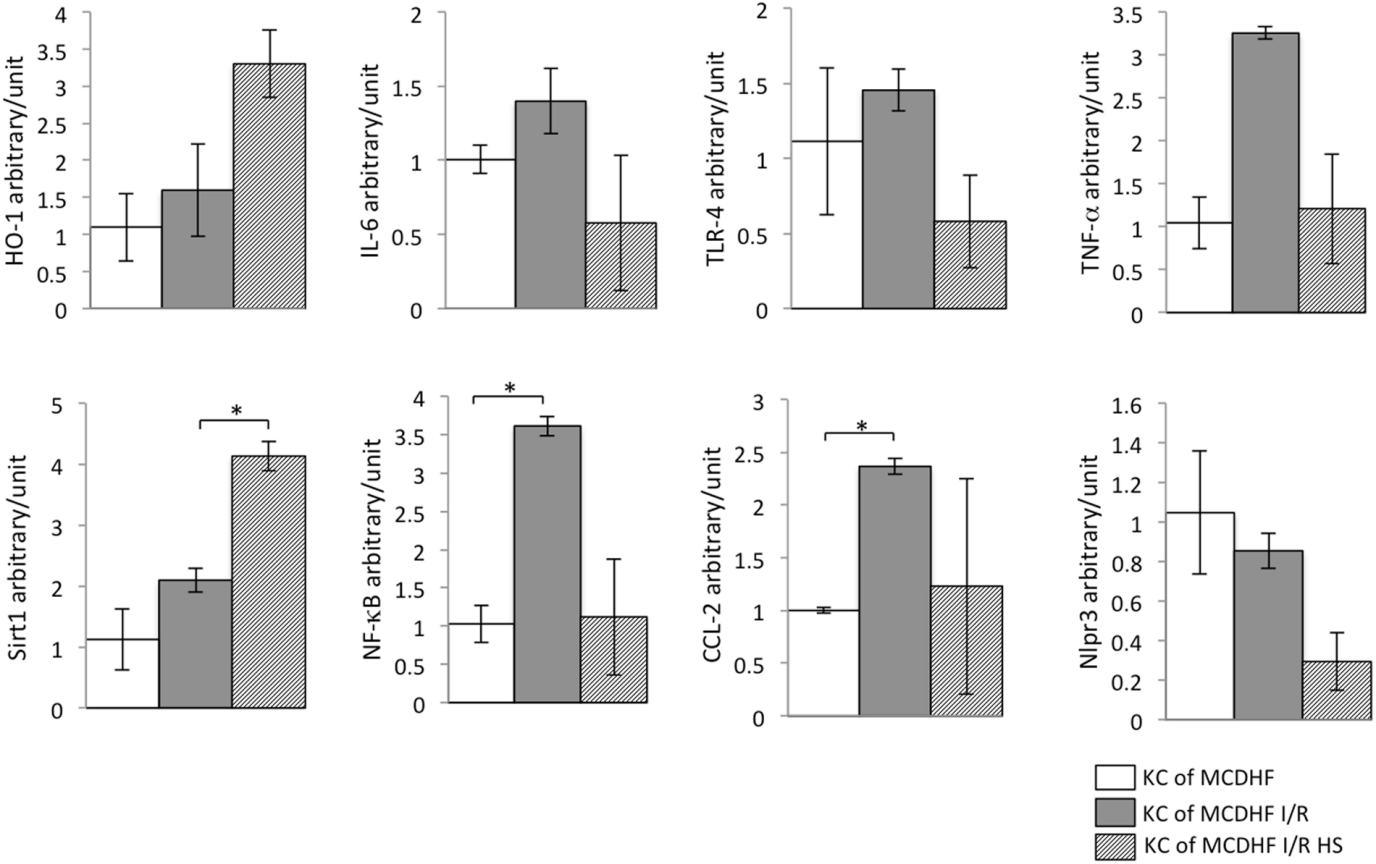
Hydrogen saline treatment induced Sirt1, HO-1 up-regulation, and the expression of inflammatory cytokine mRNA down-regulation in KCs isolated from the steatotic liver subjected to I/R. The mRNA expressions of inflammatory cytokines (TNF-α, IL-6, TLR-4, NF-κB, CCL-2), an inflammatory mediator (Nlrp3), and the expression of Sirt1 and HO-1 in KCs from fatty livers after I/R were checked by RT-PCR analysis (n = 3). Data are expressed as the means ± SEM; *p < 0.05. Data are representative of three independent experiments and indicate the mean ratio of triplicate results from each experiment.

In order to make further investigation into the role of hydrogen saline-treatment in protecting steatotic livers during I/R injury, we evaluated the inflammatory cytokine mRNA expression in KCs isolated from different groups of steatotic livers. KCs were isolated from steatotic livers subjected to 15 min ischemia and 3 hr reperfusion, pretreated or not with hydrogen saline. The KCs with hydrogen saline pretreatment showed decreased mRNA expression of inflammatory cytokines, such as IL-6 and TNF-α, more than KCs from steatotic livers subjected to I/R injury for the same time. Hydrogen saline suppressed Nlrp3. Meanwhile, the values of HO-1 and Sirt1 mRNA expression in KCs from steatotic livers were increased, especially Sirt1 was obviously up-regulated, after hydrogen saline pretreatment compared to saline treatment. In addition, we observed the mRNA expression of TLR-4 and NF-κB to further investigate the TLR-4/NF-κB signaling pathway on KCs, which were down-regulated after hydrogen saline treatment in KCs from steatotic livers after I/R injury (Fig. 3). HO-1 and Sirt1 were upregulated both in KCs and liver homogenate, confirmed by qRT-PCR analysis that indicated that hydrogen saline significantly reduced hepatic inflammation, which was induced by activation of high expression of HO-1 and Sirt1 in KCs.

### Hydrogen gas alleviated apoptosis by up-regulation of HO-1 & Sirt1 expression, corrected Bax/Bcl-2 ratio, and depressed the acetylation of p53 in steatotic hepatocytes isolated from steatotic livers subjected to hypoxia/reoxygenation

**Figure 4:**
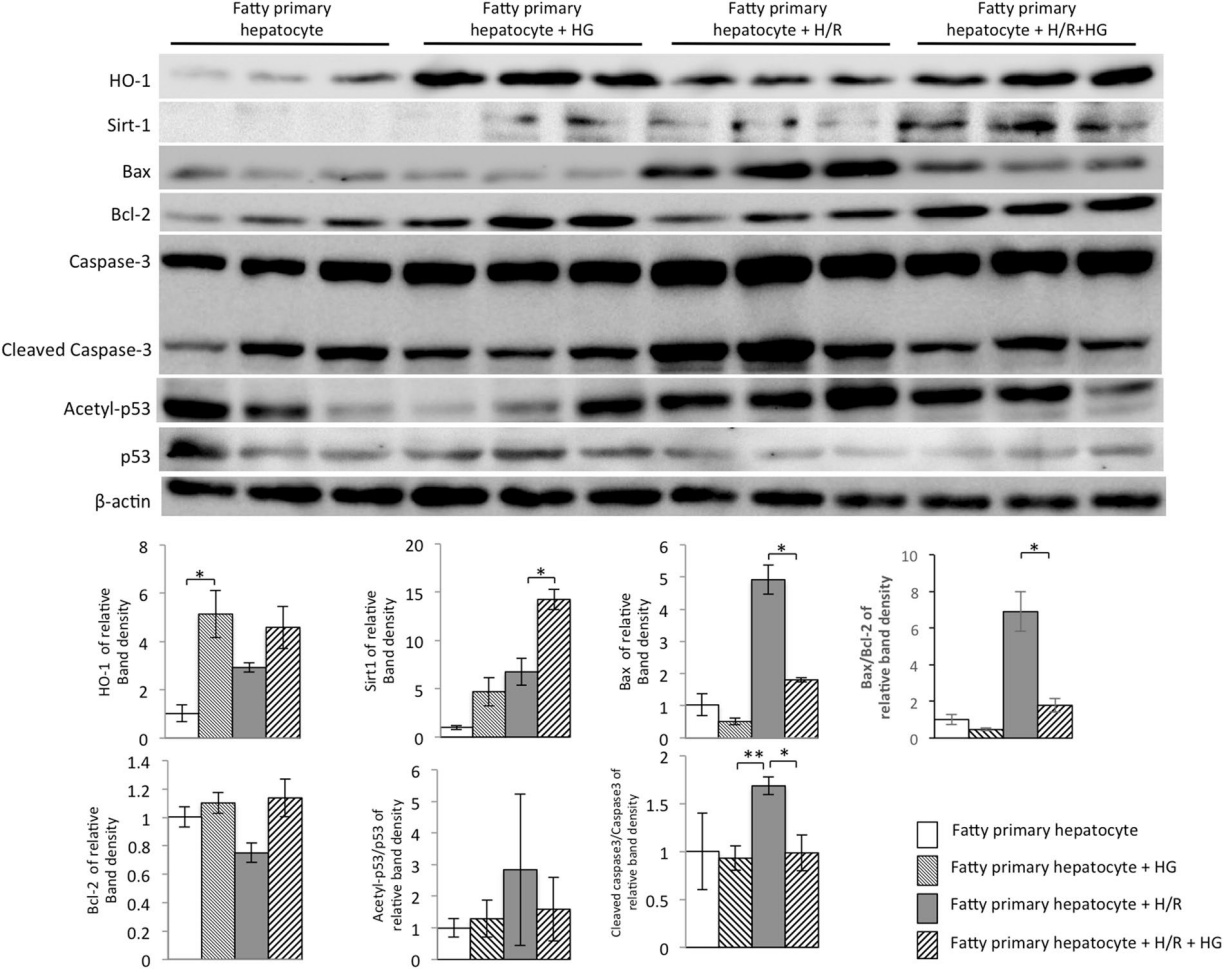
Hydrogen gas (HG) prevented steatotic primary hepatocyte apoptosis when hepatocytes were subjected to hypoxia/reoxygenation (H/R). (**A**) Expressions of Sirt1, HO-1, Bax, Bcl-2, cleaved caspase-3, and caspase-3 from steatotic primary hepatocytes after hypoxia/reoxygenation (H/R) injury which was suppressed by hydrogen gas (HG) was detected by western blot analysis. β-actin was used as a loading control. Representative blot (up) and quantified protein levels (down) are shown. Data are expressed as the means ± SEM; *p < 0.05, **p < 0.01. Data are representative of three independent experiments and indicate the mean ratio of triplicate results from each experiment.

As shown in Fig. 4, we investigated the HO-1/Sirt1 signaling pathway in steatotic primary hepatocytes after hypoxia/reoxygenation. We observed that the protein expressions of HO-1 and Sirt1 were up-regulated after hydrogen gas treatment in primary hepatocytes from steatotic livers. We observed higher expression of Sirt1 in primary hepatocytes from steatotic livers when exposed to hypoxia/reoxygenation. In addition, hypoxia/reoxygenation up-regulated the expression of Bax and slightly down-regulated or maintained almost the same level of Bcl-2 compared to the control group, whereas treatment with hydrogen gas dramatically aggravated the changes in Bax expression. The Bax/Bcl-2 ratio increased in steatotic primary hepatocytes subjected to hypoxia/reoxygenation, but significantly decreased after hydrogen gas treatment. Furthermore, there was almost 2-fold increase in the level of cleaved caspase-3 in steatotic primary hepatocytes subjected to hypoxia/reoxygenation, compared to the steatotic primary hepatocytes group. In contrast, the protein level of cleaved caspase-3 markedly decreased in the hydrogen gas treatment group.

The protein p53 can be activated through acetylation in response to apoptosis and extracellular stimulation. After hypoxia/reoxygenation, the oxidative stress reaction in steatotic hepatocytes isolated from steatotic livers is essential for apoptosis. In our study, the acetylation level of p53 was measured. The analysis results of the proportion of proteins reflected that the acetylation level of p53 in steatotic hepatocytes increased after hypoxia/reoxygenation, which was consistent with apoptosis. After administration of hydrogen gas, a decline in acetyl-p53 expression in steatotic hepatocytes was observed. All the results indicated that hydrogen gas inhibited the acetylation of p53, a critical protein responsible for mitigating cell apoptosis (Fig. 4).

## Discussion

I/R has been considered as a major, unmodifiable risk factor for fatty liver transplantation. In a a previous study^16^, we established that the MCD diet induced a hepatic steatosis model. MCD has been widely used to induce steatosis in experimental models of fatty liver. There are some kinds of diet that can induce a fatty liver mouse model, such as a high-fat diet and MCD diet. If we use a high-fat diet, steatosis and inflammation will be observed at 12–16 weeks^17^. However, in the MCD diet mouse model, fatty liver and inflammation were induced by the administration of a MCD diet in just 3 weeks. From the viewpoint of time and effect, we chose the MCD diet to establish the fatty liver mouse model. The purpose of our study was to test the protective effect of molecular hydrogen (including hydrogen-rich saline and hydrogen gas) preconditioning on I/R injury of fatty liver *in vivo* and hypoxia/reoxygenation-induced injury on primarily cultured fatty hepatocytes *in vitro*.

Molecular hydrogen acts as a novel medical application. In addition to hydrogen, nitric oxide (NO) and carbon monoxide (CO), which offer cytoprotection against cellular stress, have drawn attention^18^. The molecular hydrogen exerts cytoprotective effects in the nervous, cardiovascular, and digestive systems^19–21^. In the present study, hepatic protection by molecular hydrogen was investigated in a fatty liver mouse I/R model *in vivo* and hypoxia/reoxygenation-induced injury in a fatty hepatocyte *in vitr*o model. Notably, hydrogen saline exerted marked hepatic protection by preventing hepatocyte death and inhibiting macrophage recruitment compared with mice treated with saline alone (Fig. 1). Furthermore, its application impeded hypoxia/reoxygenation-induced damage in fatty hepatocytes (Fig. 4). During ischemia/hypoxia and subsequent reperfusion/reoxygenation, a large number of harmful substances are produced from the activated KCs, such as pro-inflammatory cytokines, and induce hepatocyte apoptosis.

KCs are considered to make an important contribution to the whole process of hepatic I/R injury. The initial stages of reperfusion produce dramatic morphological changes on activated KCs and impel them to extend into the sinusoids^22^. Activated KCs are major contributors to not only release of large amounts of cytokines, but also intracellular oxidative species (ROS)^23^. Evidence suggests that there is liver parenchymal cell injury in the hypoxic phase of the I/R injury process. The pharmacological preconditioning with the hepatic protection from pharmaceutical preparations against hepatic I/R injury by inactivation of KCs has been reported. For instance, Mosher *et al*.^24^ reported that gadolinium chloride relieves liver cell damage caused by hepatic I/R by inhibiting KC activity. In the present study, treatment with molecular hydrogen resulted in significantly increased HO-1 expression both in liver homogenate and KCs isolated from fatty liver that underwent I/R injury (Figs 2 and 3). Moreover, the proinflammatory cytokines TNF-α and IL-6 expression levels were much lower, not only in the groups with I/R injury to the liver and treated with hydrogen saline, but also in the KCs isolated from fatty liver, than those in the no-treatment groups. These data indicated that molecular hydrogen alleviated hepatic I/R injury by attenuating KC activation and possibly the expression of co-stimulatory molecules. This further confirmed the key role of KCs in the process of liver I/R injury. Moreover, this finding clearly shows that the molecular hydrogen attenuates liver I/R injury by reducing the activation of KCs, although other types of non-parenchymal cells in the liver that undergo I/R injury were not examined here.

There is growing evidence suggesting that the protective effect of molecular hydrogen is not only interrelated with simple elimination of oxygen free radicals, but also associated with various intercellular signals^25,26^. Kawamura *et al*.^27^ reported that molecular hydrogen gas ameliorated lung injury by promoting expression of Nrf2 and contributing to HO-1 expression. Cai *et al*.^28^ revealed that molecular hydrogen therapy relieved TNF-α induced rat osteoblast inflammatory injury via downregulation of the NF-κB pathway. Furthermore, there is also evidence indicating that molecular hydrogen reduced lung injury induced by transplantation by inducing elevated expression of HO-1^27^. In the present study, hydrogen saline exposure was found to markedly increase the expression of HO-1 *in vivo*. The mechanism of HO-1 in hypoxia/reoxygenation-induced hepatic insult (e.g., apoptosis) was further elucidated using *in vitro* study.

Apoptosis and necrosis are common reactions in liver when sustaining injuries induced by ischemia, radiation, and poisonous substances^29–31^. The liver and especially the hepatic parenchymal cells are sensitive to the damage caused by I/R injury^32,33^. In I/R injury, the oxidative damage to the enzyme complexes, and anti-apoptotic proteins, decrease apoptosis^34^. Inhibition of the caspase family notably attenuated liver injury caused by I/R, indicating that apoptosis plays an important role in I/R injury^35^. The hepatocyte apoptosis leads to elevated AST and ALT levels and further reduction of liver function^36^. Our investigations *in vitro* and *in vivo* support this, as seen with I/R-induced hepatocellular apoptosis by TUNEL staining (Fig. 1). The apoptotic hepatocytes were significantly increased after I/R injury, which is consistent with the results of other studies of hepatic damage after I/R^37^. The pretreatment with hydrogen saline could improve this situation. The depressed serum levels of the liver-associated transaminases ALT and AST in the group of I/R injury treated with hydrogen saline (Fig. 1) revealed that molecular hydrogen is a critical attenuator of I/R-induced hepatocyte apoptosis. A variety of molecules are involved in the regulation of apoptosis, including pro-apoptotic factor Bax and anti-apoptotic factor Bcl-2^38^. In steatosis hepatocytes sustaining hypoxia/reoxygenation injury, Bax expression was significantly increased and the expression of Bcl-2 was decreased dramatically. The hydrogen gas effectively suppressed the expression of Bax and was associated with activation of cleaved caspase-3. Meanwhile, it also promoted the expression of Bcl-2 (Fig. 4). In summary, the protective effect of molecular hydrogen against I/R was achieved by inhibiting the factors stimulating apoptosis.

**Figure 5:**
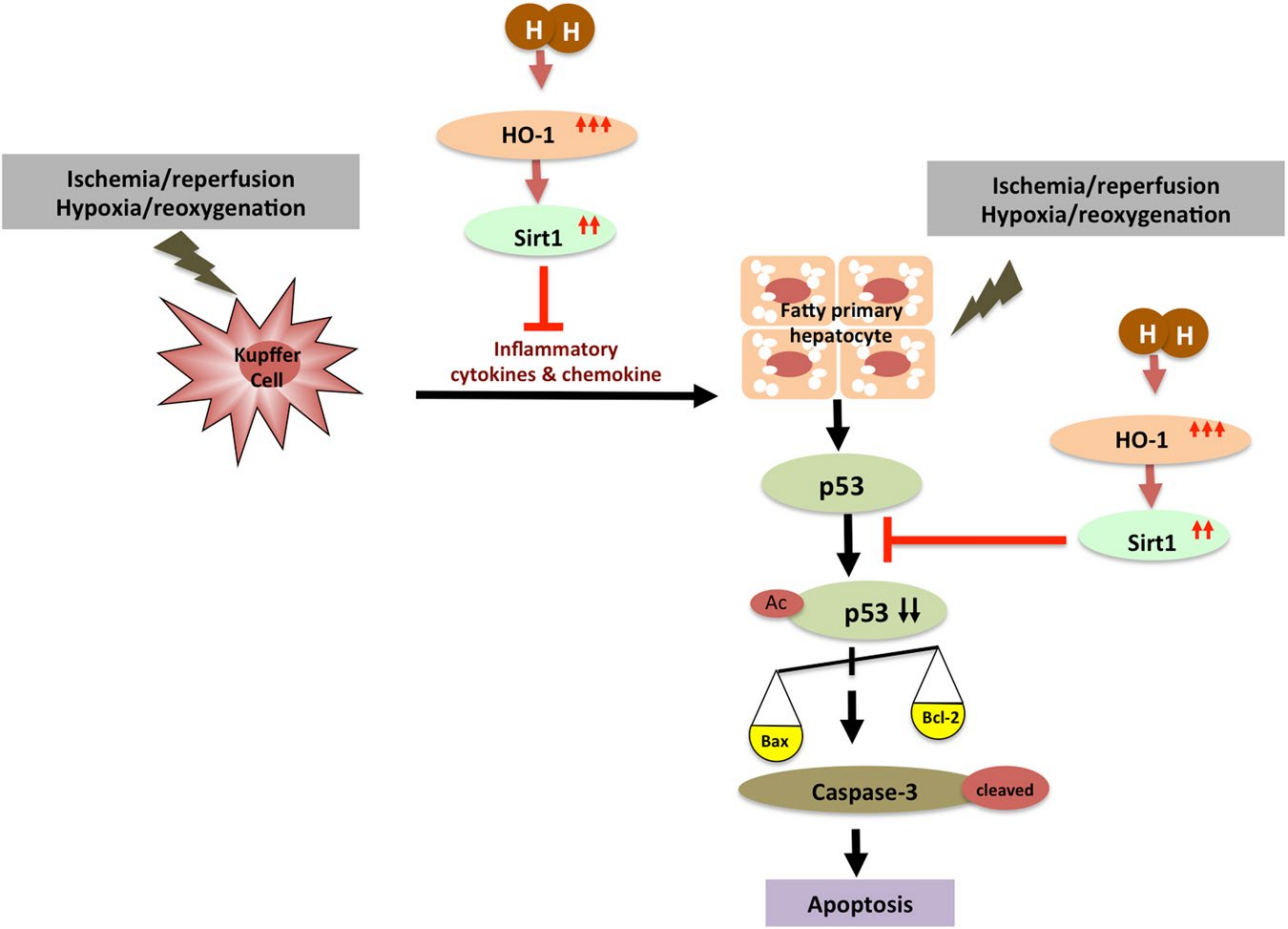
The schematic illustration of molecular hydrogen inhibiting I/R-induced liver injury. HO-1 positively regulates expression of Sirt1, while HO-1 and Sirt1 attenuates activation of Kupffer cells. Sirt1 promotes HO-1 expression and reduces the pathology of I/R or hypoxia/reoxygenation-induced hepatocyte apoptosis by repressing the deacetylating p53, balance of Bax and Bcl-2, and cleaved caspase-3.

HO-1, an important rate-limiting enzyme, contributes to heme degradation and produces biliverdin and CO. CO has anti-apoptotic and anti-inflammatory properties as well as cytoprotective effects^16^. Sirt1 is one kind of NAD-dependent protein deacetylase. It helps to regulate cytosolic metabolism and ameliorate ROS-caused apoptosis^39^. In the liver, it has been reported that Sirt1 has a hepatoprotective effect by reducing fibrosis^40^. However, the protective effect of molecular hydrogen by inducing the HO-1/Sirt1 expression in hepatocytes has not been detected until now. HO-1 is known to induce the Sirt1 expression by activating PGC-1α/ERRα^41^. Therefore, we conjectured that the anti-apoptosis effects of molecular hydrogen might be regulated by elevating HO-1-mediated induction of high expression of Sirt1. Consistent with our *in vitro* data that hydrogen gas could up-regulate the expression of Sirt1 in fatty hepatocytes, we presented the data that treatment with hydrogen saline induced the expression of Sirt1 in fatty liver. Animal studies showed that, after hydrogen saline treatment, a marked increase of HO-1 expression in the liver occurred. Furthermore, in the *in vitro* study, fatty hepatocytes exposed to hypoxia/reoxygenation exhibited an increase in the signs of apoptosis compared with normoxia; this situation was inhibited by hydrogen gas preconditioning. In addition, p53 (one of the substrates of Sirt1) is capable of binding to Sirt1 to cause deacetylation of the lysine residue in the C-terminal domain, resulting in lowering the ability of p53 to activate downstream genes, and ultimately reduces apoptosis^42^. HO-1 induces Sirt1 expression, and meanwhile, Sirt1 up-regulates tumor suppressor protein alternative reading frame (Arf) inhibited activity of p53 in macrophage-regulated hepatic sterile inflammation^43^. Our results showed that the acetylation of p53 was significantly increased in the I/R injury group, after molecular hydrogen treatment p53 acetylation was decreased, at the same time the anti-apoptotic protein Bcl-2 expression was increased, and pro-apoptotic protein Bax expression was decreased. As the data have shown, we demonstrated that the major modulator of the deacetyation of p53 is the activation of Sirt1, which in turn affects the inflammatory response and apoptosis of the affected tissue. We found that preconditioning with hydrogen gas could enhance HO-1/Sirt1 expression and reduce the expression of Bax, acetylation of p53, and cleaved caspase-3, and subsequently affect the hepatocyte apoptosis. In summary, from our data we showed a mechanism of molecular hydrogen protection against I/R-induced liver injury or hypoxia/reoxygenation-induced apoptosis of hepatocytes (Fig. 5). The protective effect of molecular hydrogen among HO-1, Sirt1, and activation of the p53 axis is by the following model. HO-1 positively regulates expression of Sirt1, while HO-1 and Sirt1 attenuates KC activation. Sirt1 inhibits activity of the p53 pathway by directly inducing apoptosis regulator Bcl-2 and by inhibiting transcription of Bax and activation of cleaved caspase-3.

Taken together, our results provide the therapeutic strategies to inhibit I/R-induced liver injury and hypoxia/reoxygenation-induced hepatocyte apoptosis via HO-1/Sirt1 activation using molecular hydrogen. Unfortunatoly this study has limination, because we use warm ischemia/reperfusion model without transplantation instead of cold ischemia/reperfusion model with transplantation. Therefore, the further examination using animal liver transplantation model should be needed to ensure the result of this study. In conclusion, molecular hydrogen may act as a potential therapeutic agent to ameliorate ischemia reperfusion injury caused by hepatic surgery or liver transplantation with steatosis.

## Materials and Methods

### Animals

Eight-week-old male C57BL/6 (B6) mice, purchased from Shizuoka Laboratory Animal Center (Shizuoka, Japan), were raised in a feeding room with automatically controlled light and temperature. Mice were cared for in agreement with the guidelines of the Institutional Animal Care and Use Committee and in agreement with National Research Institute for Child Health and Development guidelines on laboratory animal welfare.

### Grouping of animals and experimental protocol

In order to establish the steatotic liver model, mice were fed with the methionine, choline-deficient plus high fat (MCDHF) diet for 21 days. To be specific, MCDHF contains 25% oil, 20% sugar, and 55% MCD. We chose 15 min ischemia followed by 3 hr reperfusion when performing animal experiments, as described previously^44^. The mice were randomly divided into 3 groups (n = 10, each group), as follows: (1) MCDHF group:fatty liver mice with sham operation; (2) MCDHF I/R group: fatty liver mice pre-treated with saline before I/R, then sacrificed after 3 hr reperfusion; 1 ml saline spreaded on the liver after clipping hepatic portal vein with a microvascular clamp for ischemia; (3) MCDHF I/R + HS group: fatty liver mice treated with hydrogen saline before I/R, then sacrificed after 3 hr reperfusion. 1 ml 7 ppm hydrogen saline spreaded on the liver after clipping hepatic portal vein with a microvascular clamp for ischemia.

### Preparation of hydrogen saline

A solution of high-concentration, hydrogen-rich saline (7 ppm) was prepared as described previously^45^. In brief, using a hydrogen-generating agent (MiZ Co. Ltd. Kanagawa, Japan) including metallic aluminium grains and calcium hydroxide, 0.5 g of the mixture was enclosed within a nonwoven fabric and heat-sealed. Hydrogen-generating agent was inserted into a small bottle, 1 mL of pure water was added, and a cap with a check valve was placed and then tightly closed. The one-way valve bottle was inserted into a 500 mL bottle filled with 5% physiological saline. We obtained 7 ppm hydrogen saline after rocking vigorously for 5 min and storing at 4 °C for 24 hr.

### Establishment of model of hepatic ischemia reperfusion in steatotic liver

The model of hepatic I/R in steatotic liver was established according to previous works^16,44^. In particular, the steatotic liver mice ischemia was subjected by occluding the hepatic portal vein with a microvascular clamp for 15 mins followed by 3hrs reperfusion. The mice were sacrificed after 3 hr reperfusion, and the liver tissue and blood were obtained. The procedures and protocols of the experimental animals were reviewed and approved by the Committee of Care and Use of Laboratory Animals at the National Research Institute for Child Health and Development. (Permission Number: 2002-003). Further, all experiments were performed in accordance with relevant guidelines and regulations. All surgical procedures were conducted by anesthetization with isoflurane/oxygen, and all attempts were carried out to minimize suffering.

### Histopathological examination

Liver tissue was harvested for histopathological examination. In detail, the specimens of liver tissue were fixed in 10% formalin, routinely processed, and sliced into sections 4 μm thick. The severity of necrosis in steatotic liver (percentage of necrotic area) was checked by hemotoxylin-eosin (HE) staining and quantified by WinRoof V.6.1 software (Mitani Corporation, Tokyo, Japan).

### Immunohistochemical examination of tissue sections

Immunohistochemistry was performed on paraffin sections by using monoclonal antibody against F4/80 and polyclonal antibody against 4-HNE (Abcam, Cambridge, MA). After immunohistochemical staining, specimens were photographed under a microscope (Olympus, Tokyo, Japan).

### TUNEL assay

To measure hepatocyte apoptosis, the TUNEL assay was performed on liver paraffin sections to label the 3′-end of fragmented DNA by an apoptosis detection kit according to the manufacturer’s instructions (Chemicon International, Inc. Billerica, MA).

### Serum AST and ALT enzyme activity

Whole blood samples were harvested by routine processing after 15 min ischemia and 3 hr reperfusion. Serum was collected from blood samples after standing for 40 min at 25 °C and then centrifuged at 1800 × g for 20 min at 4 °C. The serum was then evaluated for AST and ALT (values expressed as U/L). AST and ALT measurements were performed by using commercially available kits (Fujifilm, Tokyo, Japan) and an automatic biochemical analyzer (DRI-CHEM 3500i, Fujifilm) according to the manufacturer’s instructions.

### RNA preparation and qRT-PCR analysis

RNA was extracted from frozen tissues using the RNeasy Mini Kit (Qiagen, Valencia, CA). Each 0.8 μg aliquot of RNA was reverse transcribed to cDNA using the Prime Script RT reagent Kit (RR037A, Takara, Japan). Quantitative RT-PCR was performed using the primer/probe sets system (the sequences are listed in Table 1) on the Applied Biosystem PRISM7900 (ABI Japan, Co. Ltd. Tokyo, Japan). The conditions of PCR cycles were 50 °C for 2 min, 95 °C for 15 min, 40 cycles of 95 °C for 30 s, 60 °C for 1 min, and 25 °C for 2 min. The ΔΔCt method was used for the relative expression, and the results were normalized by subtracting the 18 S expression.

**Table 1:** Primer sequences and probes used in this study.

Genes	Forward (5′–3′)	Reverse (5′–3′)	Probe
IL-6	CTGCAAGTGC ATCATCGTTGT	TGTCTATACCAC TTCACAAGTCGGA	CAGAATTGCCATTGC ACAACTCTTTTCTCA
TNF-α	TGTCTACTGAAC TTCGGGTGAT	AACTGATGA GAGGGAGGCCAT	TCCCCAAAGGGAT GAGAAGTTCCCAA
TLR4	CCATGCCATGCC TTGTCTT	GAGCTTCAACCC CTTGAAGATCT	TCAATTTCACACC TGGATAAATCCAGCCA
NF-κB	TGTCTGCCTCTCTCG TCTTCCT	CACCACTGTCACA GACGCTGT	CGAGGCAGCACATAG ATGAACTCCGG
18S	ATGAGTCCACTT TAAATCCTTTAACGA	CTTTAATATACGC TATTGGAGCTGGAA	ATCCATTGGAGG GCAAGTCTGGTGC
CCL2	GTTGGCTCAGCC AGATGCAG	GTAGCTCTCCAGCC TACTCATTG	CCCACTCACCTGC TGCTACTCATTCACC
HO-1	CAGGGTGACAGA AGAGGCTAAGAC	TTGTGTTCCTCTG TCAGCATCAC	TCCTGCTCAACATT GAGCTGTTTGAGGA
Sirt1	CAGCATCTTGCCTG ATTTGTAAATAC	CACCGAGGAACTA CCTGATTAAAAA	TCTCCACGAACAGC TTCACAATCAACT
NLRP3	AGGACCCACAGTGT AACTTGC	CAGAGGTCAGAGC TGAACAACA	TAAGGCCGGAATTC ACCAACCCCAGCT

### Fatty primary hepatocyte culture and establishment of the hypoxia/reoxygenation, hydrogen gas, and hypoxia/reoxygenation plus hydrogen gas conditions

Primary hepatocytes were isolated from the mouse fatty liver. Hepatocytes containing fat droplets were incubated in a 5% CO_2_ atmosphere and cultured in Dulbecco’s Modified Eagle’s Medium (DMEM, GIBCO, UK) with 10% fetal bovine serum (FBS, GIBCO). The cells were cultured at 37 °C under hypoxic conditions (1% O_2_, 5% CO_2_, and N_2_ as the base gas) in a modulator incubator chamber. Reoxygenation was obtained by replacing the cells in normal culture conditions. The hydrogen gas condition consisted of 21% O_2_, 5% CO_2_, 3.8% H_2_, and N_2_ as the base gas in a modulator incubator chamber (Billups-Rothenberg Inc. Del Mar, CA). The hypoxia/reoxygenation plus hydrogen gas condition consisted of 1% O_2_, 5% CO_2_, 3.8% H_2_, and N_2_ as the base gas for the hypoxia plus hydrogen gas phase and 21% O_2_, 5% CO_2_, 3.8% H_2_, and N_2_ as the base gas for the reoxygenation plus hydrogen gas phase; all were contained in a modulator incubator chamber. After adjusting the concentration of gas, the modulator incubator chamber was placed in a 37 °C incubator.

### *In vitro* experimental design

The primary hepatocytes bearing fat droplets isolated from steatotic liver were randomly divided into four groups as follows:

Group 1: Control group, fatty primary hepatocytes;

Group 2: Hydrogen gas group, fatty primary hepatocytes treated 10 hr with hydrogen gas;

Group 3: Hypoxia/reoxygenation group, fatty primary hepatocytes subjected to 4 hr of hypoxia and 6 hr of reoxygenation;

Group 4: Hypoxia/reoxygenation + hydrogen gas group, fatty primary hepatocytes treated with hydrogen gas plus hypoxia/reoxygenation.

### Effects of hydrogen saline on inflammation and cytotoxicity in KCs isolated from steatotic liver after I/R injury

Perfusion solution was delivered to the portal vein of the anesthetized steatotic mouse liver, then the inferior vena cava was cut immediately to establish the perfusion circulatory pathway, and the speed was set at 3 mL/min. Collagenase solution replaced the liver perfusion solution after 5 min perfusion, and the collagenase solution perfusion continued for 8 min at the same speed. After collagenase continued the digestion, the liver was removed to a sterilized 100 mm culture dish, and then was ground to form a homogenate. The liver homogenate was re-suspended in culture medium, and passed through a 70 μm filter for removing undigested clots.

Cell suspensions were centrifuged at 720 × g (Beckman Coulter Allegra X-12R, Indianapolis, IN) for 7 min at 4 °C. The top aqueous phase was discarded and the cell sediments were reserved. Cell sediments were re-suspended in 30 mL DMEM and centrifuged at 720 × g for 7 min at 4 °C, the aqueous phase was discarded, and the cell sediments were reserved. Cell sediments were re-suspended in 10 mL GBSS-B and mixed with 14 mL GBSS-A containing Nycodenz, and then centrifuged (1900 × g, 20 min, 4 °C). Harvested cells of the white layer were re-suspended in 30 mL GBSS-B and centrifuged (50 × g, 7 min, 4 °C). The top aqueous phase was transferred into a 50 mL tube and centrifuged (720 × g, 7 min, 4 °C). The cell sediments were seeded into a 6-well plate at the density of 1 × 10^7^/well in DMEM medium with 10% FBS and 100 U/mL penicillin/streptomycin and incubated in a 5% CO_2_ incubator at 37 °C. Four hours later, non-adherent cells were removed by washing twice with PBS; the adherent cells were KCs.

The total RNA was extracted from KCs, reverse transcribed to cDNA, and detected by qRT-PCR.

### Effects of hydrogen gas on anti-apoptosis in fatty hepatocytes isolated from steatotic liver subjected to hypoxia/reoxygenation

The method of liver perfusion and digestion was the same as for the KCs. The liver homogenate was re-suspended in culture medium, and passed through a 100 μm filter for removing undigested clots. Cell suspensions were centrifuged at 80 × g (Beckman Coulter Allegra X-12R) for 1 min at 4 °C. The top aqueous phase was discarded and the cell sediments were reserved. Cell sediments were re-suspended in 30% Percoll and centrifuged at 80 × g for 5 mins at 4 °C, the aqueous phase was discarded, and the cell sediments were reserved. Cell sediments were re-suspended with 45% Percoll and centrifuged at 80 × g for 2 mins at 4 °C. The top aqueous phase was mixed with 2 folds of DMEM and transferred to a 50 mL tube and centrifuged (80 × g, 1 min, 4 °C). The cell sediments were seeded into a 6-well plate at the density of 1 × 10^6^/well in DMEM medium with 10% FBS and 100 U/mL of penicillin/streptomycin and incubated in a 5% CO_2_ incubator at 37 °C. Twenty-four hours later non-adherent cells were removed by washing twice with PBS; the adherent cells were fatty hepatocytes. Then the total RNA was extracted from fatty hepatocytes, reverse transcribed to cDNA and detected by qRT-PCR. The protein of fatty hepatocytes was disintegrated by radioimmunoprecipitation assay (RIPA) lysis buffer and stored in liquid nitrogen for western blot analysis.

### Western blot analysis

Proteins from fatty hepatocytes were homogenized with RIPA lysis buffer (Wako, Osaka, Japan) containing phosphatase inhibitor cocktail and protease inhibitor cocktail. Proteins were blotted onto PVDF membranes (Bio-Rad, Hercules, CA). The membranes were incubated with appropriate primary antibodies overnight at 4 °C after being blocked with skim milk for 1 hr at room temperature. Afterwards, the membranes were washed three times with TBST and incubated with secondary antibodies for 1 hr at room temperature. The blots were visualized by enhanced chemiluminescence. The primary antibodies were as follows: HO-1 (1:2000; Abcam), Sirt1 (1:1000; Cell Signaling Technology), Bax (1:1,000; Santa Cruz Biotechnology), Bcl-2 (1:1000; Cell Signaling Technology), Cleaved Caspase-3 & Caspase-3 (1:1000; Cell Signaling Technology), Acety-p53 & p53 (1:1000; Cell Signaling Technology), β-actin (1:2000; Cell Signaling Technology).

### Statistical analysis

Data were presented as means ± SEM (means with standard errors) and analyzed statistically by a one-way ANOVA followed by Student’s *t*-test. A value of *p* < 0.05 was considered to be statistically significant.
